# PD167 Charting A Sustainable Way To Subsidize Implants In Singapore

**DOI:** 10.1017/S0266462324003933

**Published:** 2025-01-07

**Authors:** Swee Sung Soon, Wayne Ding, Yen Ee Wong, Yan Teck Ho, Lydia Ooi, Rachel See-Toh, Ada PM Batcagan-Abueg, Jun Yi Ho, Foo Eng Low, Bindhu Unni, Hua Pey Gan, Chong Ye Yeo, Jeshuan Heng, Sarah Chew, Qinyi Lim, Elysia Lim, Zhen Long Ng, Jaryl Ng, Hong Ju, Kwong Ng

## Abstract

**Introduction:**

The diverse implant landscape, the rising and disparate costs of implants in public healthcare institutions (PHIs), and the limited application of health technology assessment (HTA) impede fair and sustainable implant subsidies in Singapore. This study described the Agency for Care Effectiveness (ACE) Implant Subsidy List (ISL) methodology and the key enablers for supporting government subsidy of clinically effective and cost-effective implants in Singapore.

**Methods:**

A multi-tiered implant grouping scheme on the ISL was established by adapting overseas implant classifications, consulting clinicians, and conducting HTA evaluations, with subsidy extensions at the product group tier. Implants within a product group share similar biomechanical actions and patient outcomes and are subject to the same clinical criteria and pricing requirement. Implants on the ISL must be approved by the regulatory authority. Patients who meet the clinical criteria for ISL implants are eligible for subsidy. ACE conducted value-based pricing (VBP) and partnered with the public healthcare supply chain agency to harmonize PHI implant prices. The ISL is updated three times per year.

**Results:**

Implants listed on the ISL were deemed clinically and cost effective. Underpinned by HTA principles, the implant grouping scheme promoted parsimonious classification, while allowing the creation of new product groups for implants offering superior benefits for patients. Reasonable prices set for the product groups aided affordability and cost sustainability. The ISL clinical criteria and standardized implant identifiers encouraged the appropriate use of subsidized implants and facilitated implementation. By ISL implementation in December 2023, ACE assessed 42,165 implants and listed 22,689 ISL implants spanning 143 product groups. Industry can apply for ISL listing three times per year, which keeps the ISL updated and relevant.

**Conclusions:**

The ISL adopts a fit-for-purpose methodology to standardize implant classifications, enable scalable application of HTA, drive appropriate use of subsidized implants, and bring cost sustainability to the government subsidy of implants in Singapore. A strategic partnership with the public healthcare supply chain agency to concurrently establish national procurement contracts reduced disparate implant prices in PHIs and provided greater leverage for better implant prices.

